# Editorial: Molecular Mechanisms in Chronic Kidney Disease

**DOI:** 10.3389/fcell.2021.712834

**Published:** 2021-06-29

**Authors:** Claudia Torino, Evangelia Dounousi, Samar Abd ElHafeez, Nejra Prohic

**Affiliations:** ^1^Institute of Clinical Physiology, National Research Council, Reggio Calabria, Italy; ^2^School of Health Sciences, University of Ioannina, Ioannina, Greece; ^3^High Institute of Public Health, Alexandria University, Alexandria, Egypt; ^4^Clinical Center University of Sarajevo, Sarajevo, Bosnia and Herzegovina

**Keywords:** CKD, ESRD, aetiology, biomarkers, risk factors

Recent progress in the field of medicine, together with improved therapeutic resources, resulted in a significant lengthening of the life span, with the consequent increase in the prevalence of chronic diseases, typically associated with age. Among the non-transmissible diseases, Chronic Kidney Disease (CKD), represents a major public health problem, having reached a worldwide prevalence of 13% (Hill et al., 2016).

Molecular mechanisms underlying CKD have been investigated for long, unravelling the role of membrane proteins, inflammatory processes or even microbiota in the development and progression of this disease (Figure 1).

**Figure 1:**
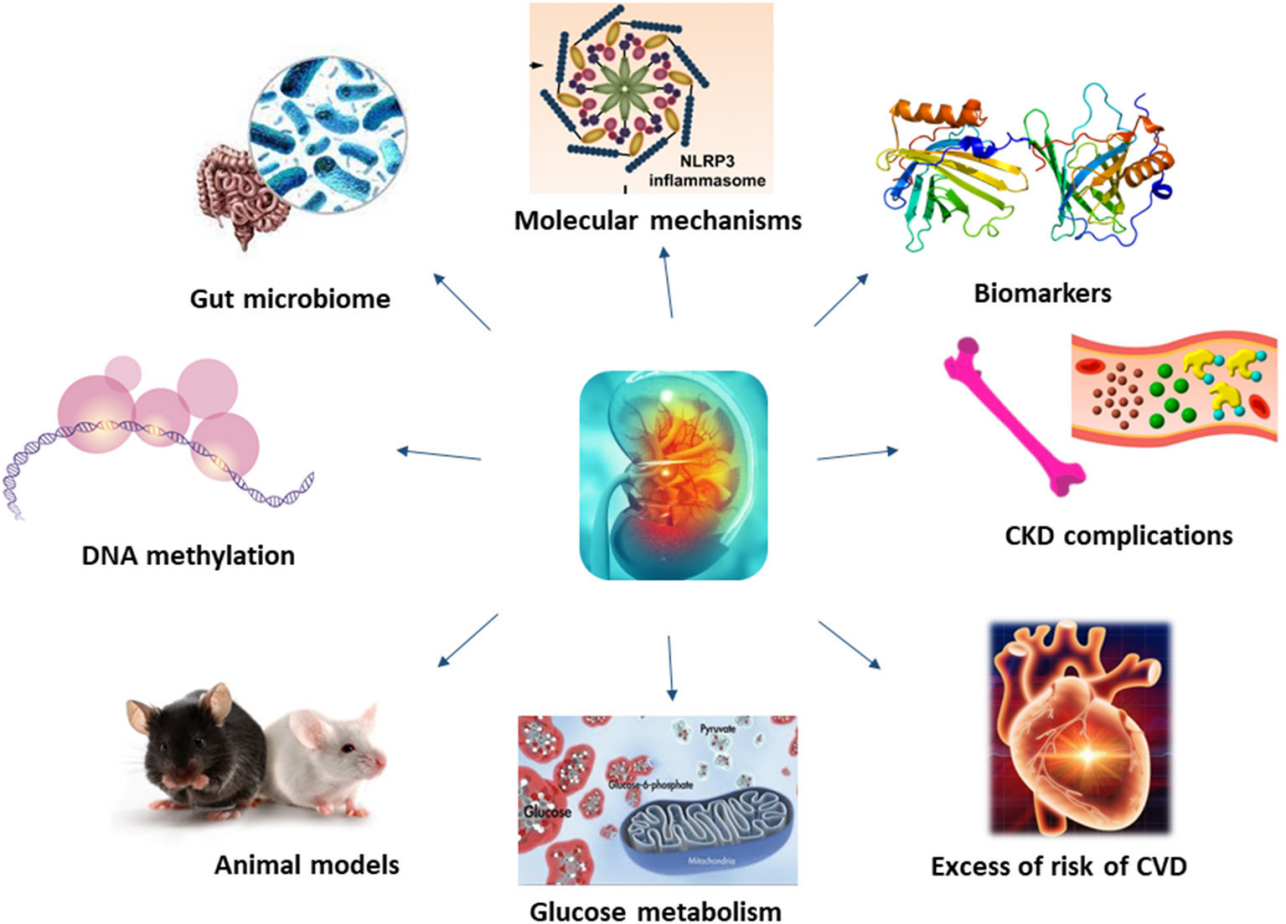
Molecular mechanisms underlying CKD have not been fully understood. Recent studies, thanks to the application of new technology and more efficient animal models, have shed light on novel mechanisms and biomarkers of CKD, also unravelling the role of kidney on glucose metabolism, CKD and CVD complications.

One example is given by Nod-like Receptor Pyrin domain containing proteins (NLRPs), expressed by resident renal cells. Rossi et al. investigated the possible role of NLRPs in cystinosis, a genetic disorder that affects kidney proximal tubular epithelial cells (PTEC). Analysing human control and cystinotic immortalised PTEC, they found that cystinotic cells had higher expression levels of NLRP2, suggesting that NLRP2 may have a key role in regulating proinflammatory, profibrotic, and antiapoptotic responses in PTEC, contributing to the pathogenesis of this disease. Focusing on inflammation, NLRP3 inflammasome activation plays a central role in renal damage. This process is negatively regulated by autophagy, investigated by Wang et al. in order to identify potential therapeutic approaches. Analysing the effect of pterostilbene (PT) on renal fibrosis, they found a significant reduction of renal fibrosis in CKD models, reduction due to the downregulation of NLRP3 inflammasome activation. Another mechanism involved in kidney fibrosis is the epithelial–mesenchymal transition (EMT). As the Transient Receptor Potential Channel 6 (TRPC6) knockout plays an anti-fibrotic role, Zhang et al. investigated this relationship in mice models. Limiting the search on diabetic nephropathy, O-GlcNAcylation, a post-translational modification of proteins, may be involved in the pathogenic process. In order to unravel mechanisms downstream this phenomenon, Costa et al. investigated his role in the impairment of the regulatory volume decrease (RVD) after cell swelling. Their study shows that O-GlcNAcylation affects RVD by influencing IClswell, i.e., the chloride current induced by cell swelling.

The recent advances in sequencing technologies have notably increased our knowledge of the gut-kidney axis. In their paper, Liu et al. explored the possible link between short-chain fatty acids (SCFAs), diet and gut microbiome, finding a partial protection against AKI and subsequent CKD in mice fed with a high content of fibre. This led to the conclusion that dietary manipulation of the gut microbiome protects against kidney disease onset and progression.

Among the new frontiers of genetics, epigenetics investigates modifications in gene expression (i.e., methylation) without changing the DNA sequence. Smyth at al. analysed DNA methylation in 677 diabetic patients with or without DKD, finding high methylation levels in 2 genes involved in RNA transcription and splicing (ZNF187 and CCNL1).

One of the difficulties in unravelling molecular mechanisms of CKD is that results obtained in animal models may diverge from that obtained in real patients. Nephronophthisis (NPH), an autosomal recessive ciliopathy, is an example. In their review, Stokman et al. summarise the most important findings of therapeutic approaches in NPH model systems. Looking at data available so far, in spite of many potential therapies identified, results are limited by the lack of animal models able to reproduce the juvenile NPH phenotype. Employment of non-orthologous animal models and developments in organoid technology can potentially fill this gap and provide new opportunities for personalised treatments. Better results were obtained with diabetic nephropathy (DN). Liu et al. explored its pathogenesis in two different animal models, finding that a spontaneous T2DM model, established by feeding KK-Ay mice with a high-fat diet, showed renal dysfunction and pathology before mice in which diabetes was chemically induced, with increased in severity over the length of the experiment. As KK-Ay mice showed earlier onset of the typical pathological characteristics associated with T2DM, together with renal lesions suggestive of kidney damage, they could be useful preclinical model for the development of anti-DN drugs. An example of therapeutic approach developed in animal models is given by Wang et al., who tested the efficacy of icariin, the flavonoids used in traditional Chinese medicine for kidney disease. They results showed an improvement of renal parameters, as well as positive histopathological changes of kidney, suggesting that the treatment could inhibit T1DM.

If effects of glucose on kidney are clear, kidney function in glucose metabolism is less known. In this review, Pina et al. summarise literature available on insulin and kidney, focusing on kidney dysmetabolism and hyperinsulinemia/insulin resistance (IR). It emerges a new role for kidney: not only a mere target of insulin action, but also damaged by IR, even in absence of diabetes. However, if IR is responsible for the onset and progression of CKD, or vice versa, need to be clarified.

Whilst some of the molecular mechanisms resulting in renal failure have been unravelled, those underlining CKD complications are more complex, being associated with coagulation abnormalities, endothelial dysfunction, vascular calcification, uremic toxins, oxidative and metabolic stress and inflammation (Culleton et al., 1999; Schillaci et al., 2001; De Leeuw et al., 2002; Anavekar et al., 2004; Shlipak et al., 2004; Weiner et al., 2004) (Figure 1). Among uremic toxins, those tryptophan-derived toxins are of particular interest because of their cardiovascular toxicity and its bound with the aryl hydrocarbon receptor (AhR), considered a potential target for new therapeutic interventions. In their review, Mo et al. showed the existing link between AhR and CKD complications, including cardiovascular disease (CVD), and its involvement in the enhancement of the intestinal barrier function to mitigate the effects of uremic toxins.

Among biomarkers potentially useful for monitoring kidney disease, Neutrophil Gelatinase-Associated Lipocalin (NGAL), holds promising capacities in predicting renal function worsening in various renal diseases. Starting from this hypothesis, Coppolino et al. tested the efficacy of urinary NGAL (uNGAL) in 61 patients affected by glomerulonephritides (GNs). They found that uNGAL levels were inversely correlated with eGFR, with progressor subjects showing exceedingly high baseline uNGAL values as compared with non-progressors. Furthermore, subjects with uNGAL values above the optimal cut-off of 107 ng/mL experienced a more rapid progression to the renal endpoint, indicating that this biomarker may represent a real-time indicator of renal damage and an independent predictor of renal disease progression. Another biomarkers thought to be involved in the pathogenesis and development of kidney disease is soluble urokinase plasminogen activator receptor (suPAR). Iversen et al., analysing retrospectively data from 25,000 patients, found that elevated suPAR was independently associated with incident chronic and acute kidney conditions, highlighting the potential for using suPAR in risk classification models to identify high-risk patients who could benefit from early clinical interventions.

CKD progression is strongly influenced by diabetes, hypertension, and CVD. Even though it is known in literature that hypomagnesemia is associated with the development and progression of these comorbidities, no indications on the usefulness of Mg supplementation in preventing adverse clinical outcomes are available. In their analysis, Rodelo-Haad et al. conclude that no strong evidence is available to make a formal recommendation to prescribe Mg supplements. However, some data strongly suggest the benefits of Mg on vascular calcification, CVD, and kidney function, indicating that Mg supplementation, if indicated, may slow down the development of such negative morbidities.

Patients with CKD have an almost uniquely high risk of death and cardiovascular disease, and with a rate of incident cardiovascular events strongly associated with the level of renal function (Zoccali, 2006; Figure 1). Contrarily to the general population, where as much as the 75% of excess risk for coronary heart disease could be explained by classical risk factors (Magnus and Beaglehole, 2001), the excess of risk of CVD in CKD patients it is not so easy to explain. Furthermore, as renal function deteriorates, the risk increases linearly (Weiner et al., 2004). To this proposal, Provenzano et al. report, in their review how albuminuria and eGFR reduction predict higher CV risk, independently from traditional risk factors. Furthermore, the addition of eGFR and albuminuria to risk prediction models including the traditional CV risk factors (i.e., Framingham risk score) improved the predictive accuracy, leading to consider CKD as an equivalent CV risk.

Among novel risk factors for complications in CKD, in their review Georgatzakou et al. focused on extracellular vesicles (EVs), membrane-enclosed nanoparticles released by most cells in body fluids and extracellular matrix, whose function is signal transduction. Plasma EVs may have significant roles in CKD as disease biomarkers, risk factors for the development of complications, repair or protective factors diffusing harmful stress signals, and finally, therapeutic factors. Development of therapeutic interventions have already been oriented to both block their negative effects and take advantage of their beneficial properties. EVs, as promoters of vascular damage and subsequent development of CVD, are also described in the review by Carracedo et al., who discussed several aspects that characterise CKD-associated CVDs, such as etiopathogenic elements that CKD patients share with the general population, changes in the cellular balance of reactive oxygen species (ROS), and the associated process of cellular senescence. In particular, they reported how uremia-associated ageing is associated to numerous changes both at the cellular and molecular level, changes similar to those observed in the normal process of physiologic ageing. Understanding the processes and factors involved in accelerated senescence and other abnormal intercellular signalling could lead to identify new therapeutic targets and lead to improved methods of diagnosis and monitoring for patients with CKD-associated CVDs.

CKD patients often present alterations in the bone and mineral metabolism, and in dialysis patients the existing association between bone fractures and cardiovascular comorbidities is well-known. Paget bone disease (PDB) is a cause of osteoporosis and bone fragility and exposes patients to a high incidence of bone fractures but, as secondary hyperparathyroidism is a frequent condition in dialysis patients, it can easily mask PDB diagnosis. As reported by Panuccio and Tripepi, there are limited reports on the treatment of patients with CKD and PBD. Furthermore, management of these patients is a challenge because these diseases act synergistically in affecting bone quality and increasing the risk of fractures. According to the 2017 KDIGO guideline, an aggressive control of serum parathyroid hormone, calcium, phosphorus, and vitamin D level for the CKD-MBD patient is strongly suggested. It is important to individualise therapy and to try to prevent falls with regular exercise to maintain joint mobility and bone strength.

Alterations in the bone and mineral metabolism, typical of CKD, has a direct consequence the onset of vascular calcification induced by hyperphosphatemia. Lanthanum drugs are commonly used ad phosphate binders in CKD patients, so Zhao et al. investigated, in animal models, their potential efficacy in the treatment of vascular P-induced calcifications in CKD patients. Comparing differently treated rats, authors found an improvement in biochemical and histopathological parameters in the lanthanum but not in the control group, indicating the efficacy of lanthanum hydroxide in postponing CRF progression and in protecting renal function. In addition, lanthanum hydroxide postponed hyperphosphatemia-mediated vascular calcification in CKD via the NF-κB signal transduction pathway, suggesting a protection against renal failure and high phosphorus level, thus postponing vascular calcification progression.

Understanding mechanisms underlying CKD is fundamental to develop novel treatments for this disease and its complications (Figure 1). In recent years, bioinformatics has emerged as a powerful tool for providing comprehensive insights into the molecular mechanisms of disease and identifying potential biomarkers and therapeutic targets. Novel methods, such as bioinformatics analyses applied to genome and proteome, might help in identifying new, unknowns, pathways.

## Author Contributions

CT drafted the initial manuscript. All authors revised, approved the final version, and agree to be accountable for the content of the work.

## Conflict of Interest

The authors declare that the research was conducted in the absence of any commercial or financial relationships that could be construed as a potential conflict of interest.
